# Assessment of Heat Hazard during the Polymerization of Selected Light-Sensitive Dental Materials

**DOI:** 10.1155/2016/4158376

**Published:** 2016-10-20

**Authors:** Maciej Janeczek, Katarzyna Herman, Katarzyna Fita, Krzysztof Dudek, Małgorzata Kowalczyk-Zając, Agnieszka Czajczyńska-Waszkiewicz, Dagmara Piesiak-Pańczyszyn, Piotr Kosior, Maciej Dobrzyński

**Affiliations:** ^1^Department of Biostructure and Animal Physiology, Wroclaw University of Environmental and Life Sciences, Kożuchowska 1, 51-631 Wroclaw, Poland; ^2^Department of Conservative Dentistry and Pedodontics, Wroclaw Medical University, Krakowska 26, 50-425 Wroclaw, Poland; ^3^Faculty of Mechanical Engineering, Technical University of Wroclaw, Łukasiewicza 5, 50-371 Wroclaw, Poland

## Abstract

*Introduction.* Polymerization of light-cured dental materials used for restoration of hard tooth tissue may lead to an increase in temperature that may have negative consequence for pulp vitality.* Aim.* The aim of this study was to determine maximum temperatures reached during the polymerization of selected dental materials, as well as the time that is needed for samples of sizes similar to those used in clinical practice to reach these temperatures.* Materials and Methods.* The study involved four composite restorative materials, one lining material and a dentine bonding agent. The polymerization was conducted with the use of a diode light-curing unit. The measurements of the external surface temperature of the samples were carried out using the Thermovision®550 thermal camera.* Results.* The examined materials significantly differed in terms of the maximum temperatures values they reached, as well as the time required for reaching the temperatures. A statistically significant positive correlation of the maximum temperature and the sample weight was observed.* Conclusions.* In clinical practice, it is crucial to bear in mind the risk of thermal damage involved in the application of light-cured materials. It can be reduced by using thin increments of composite materials.

## 1. Introduction

Light-cured materials are commonly used in dental treatment. The polymerization process takes place by activating a photoinitiator present in dental resin by using a light-curing unit. At present, a quartz-tungsten halogen (QTH) or a light-emitting diode (LED) light-curing units are used. Less popular solutions include the plasma-arc-source-PAC or the argon laser. Halogen lamps emit waves which are 360–560 nm in length, and the radiation flux density, also called light intensity of the majority of devices, ranges from 700 to 800 mW/cm^2^ although in some cases it exceeds 1500 mW/cm^2^. When compared with QTH, LED light-curing units are more efficient and they use less energy. The older generation of diode photopolymerization units emit light of wavelengths of approximately 468 nm which is of central importance for classical photoinitiators such as camphorquinone which are contained in the materials. The newer generation of LED (polywave) emits wavelengths of wider spectra (from 460 to 410 nm), as a result of which it is also effective for alternative photoinitiators [1–3].

Undoubtedly, introducing light-cured materials in clinical practice was a milestone in dentistry. However, these materials are not flawless. One of their drawbacks is the polymerization shrinkage which may result in marginal leakage of the restoration. In order to minimize the risk of the shrinkage, the composition of the materials is modified, special application techniques are introduced, and alternative light-curing techniques are recommended; for example, soft-start, pulsation, or pulsation soft-start are used. Another significant issue is a temperature increase during polymerization process. On the one hand, it results from the curing unit emitting light energy; on the other hand, it is linked with the heat generated during the exothermic reaction [2, 4, 5]. It has been established that dentine has good insulation properties thanks to which the pulp tissue is protected from overheating [6]; however, many researchers emphasize the risk of its occurrence, particularly in deep cavities [7–12]. A temperature increase of 5.5°C may already cause tissue damage [13]. It has been determined that the threshold temperature value at which irreversible disturbances of the pulp circulation are initiated is 42.5°C [14, 15]. Therefore, it can be speculated that speed and duration of thermal stimulus as well as the extent of temperature rise play an important role in pulp damage and gradual temperature increase may raise the threshold temperature higher than 5.5°C.

The measurements of the temperature changes which occur in the light-cure materials during the polymerization process can be carried out by applying various methods, for example, using thermocouples [16–18] or thermography [19, 20]. The values of the temperature changes observed by different authors varied considerably, since they depended not only on the method of measurement but also on many other factors, such as the location of the measurement, the type and shade of the material, and the thickness of the light-cured increment.

The aim of this study was the comparative assessment of the maximum temperature rise and the duration of polymerization process of selected light-cured dental materials, as well as the analysis of the influence of their volume on thermal parameters. The hypothesis of this study predicts that the dangerous increase in temperature during the polymerization of light-cure composite materials has negative consequence for pulp vitality.

## 2. Materials and Methods

The following light-sensitive dental materials were involved in the study:

*Composite Restorative Materials*

- Te-Econom (hybrid, Ivoclar Vivadent, Schaan, Liechtenstein)
- Filtek Supreme XT (nanofil, 3M ESPE, St. Paul, MN, USA)
- Tetric EvoCeram (nanohybrid, Ivoclar Vivadent, Schaan, Liechtenstein)
- Gradia Direct (microhybrid, GC Corp, Tokyo, Japan)

*Lining Material*

- Ionosit (compomer, DMG, Hamburg, Germany)

*Bonding Agent*

- ExciTE (V generation, Ivoclar Vivadent, Schaan, Liechtenstein).

**Figure 1:**
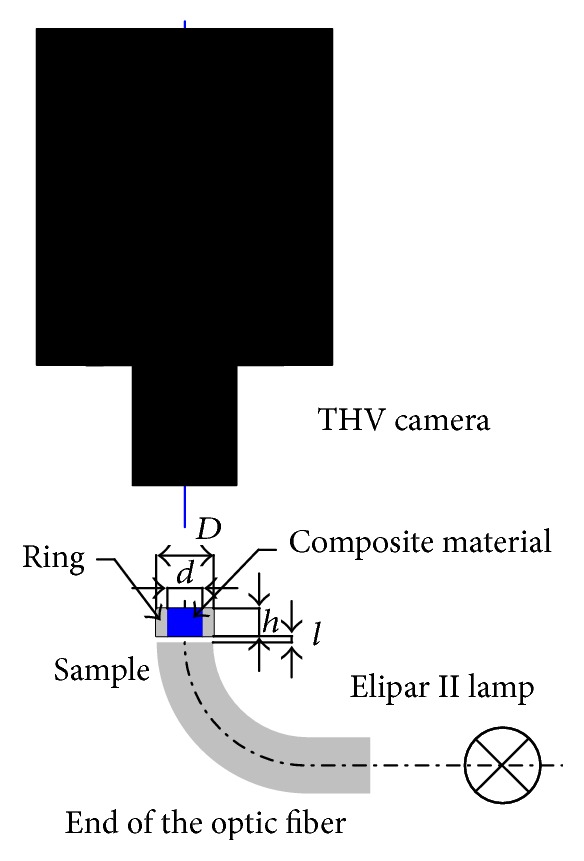
Measurement system diagram.

The complete list of materials of various translucency and color intensity, as well as the size of the samples and polymerization conditions, is shown in Table 1. The research was conducted with the use of the Elipar II LED (3M ESPE) light-curing unit that emits waves of 400–515 nm in length, with the maximum light intensity of 800 mW/cm^2^. The composite materials were light-cured by the polymerization unit for 40 s, lining material −20 s, and bonding agent −10 s. Samples of dental restorative materials were prepared in form of cylinders which filled Teflon rings of various height (*h*) and internal diameter (*d*) and the external *D* = 10 mm (Figure 1). Samples of bonding agents and lining materials were placed on 0.5 mm thick transparent PC plates with the use of a pipette. Constant temperature measurement of the external surface of the samples was carried out using the Thermovision 550 thermal camera in an isolated, dark, and air-conditioned laboratory. The measurements were taken at a distance of 45 mm with the use of a wide-angle lens and a distance ring. As a result, thermograms in which samples fulfilled approximately 35% of the picture area were obtained.

**Figure 2:**
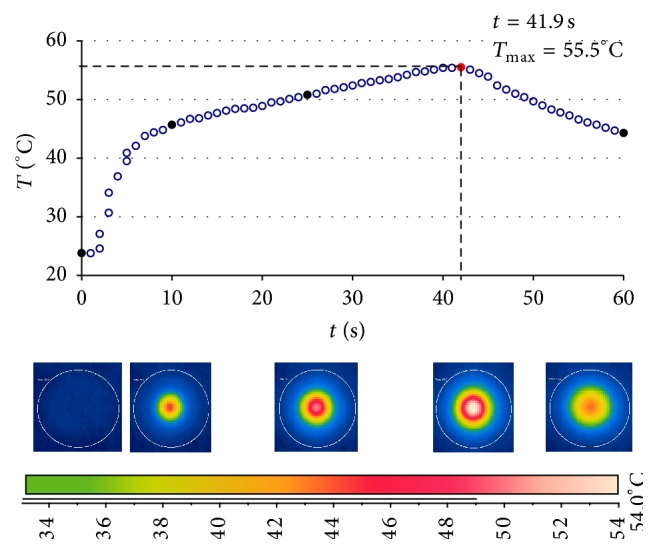
Example of temperature change in the Gradia A-3 sample (*m* = 170 mg; *h* = 2 mm; *d* = 8 mm).

The prepared samples were placed at an *l* distance from the tip end of the lamp optical fiber which was placed opposite the lens of the thermal camera. The *l* value was 0 or 3 mm. After the temperature of the sample has set at the level of approximately 24°C, the light-curing unit initiating the polymerization process and the program managing the acquisition of data from the thermal camera were simultaneously activated [21]. The measurements of the heat generated in the polymerization process were taken until the maximum temperature of the sample fell below 30°C (Figure 2).

The strength and direction of the relationship of the maximum temperature reached by the samples in the polymerization process, as well as their weight, were determined by calculating the value of the *r* Pearson correlation coefficient. The Kruskal-Wallis test was used to verify the hypothesis of lack of significant differences between the maximum temperature *T*_max_ and the time of reaching *t*_max_ and the type of material. The calculations were made with the use of the STATISTICA version 12 software.

## 3. Results

**Figure 3:**
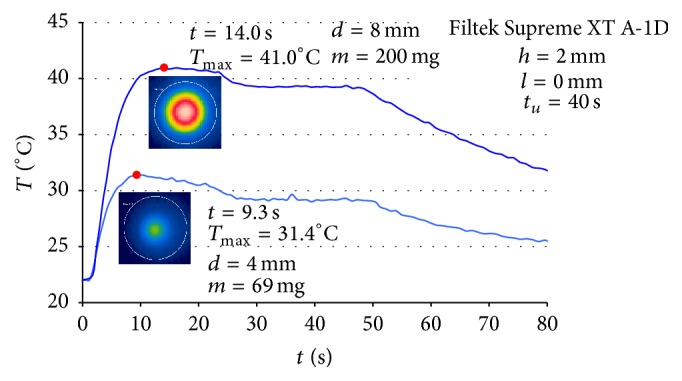
Example of temperature change during the polymerization of samples of Filtek Supreme XT A-1D of various weight and the time of exposure to the light of the light-curing unit: *t*_*u*_ = 40 s.

**Figure 4:**
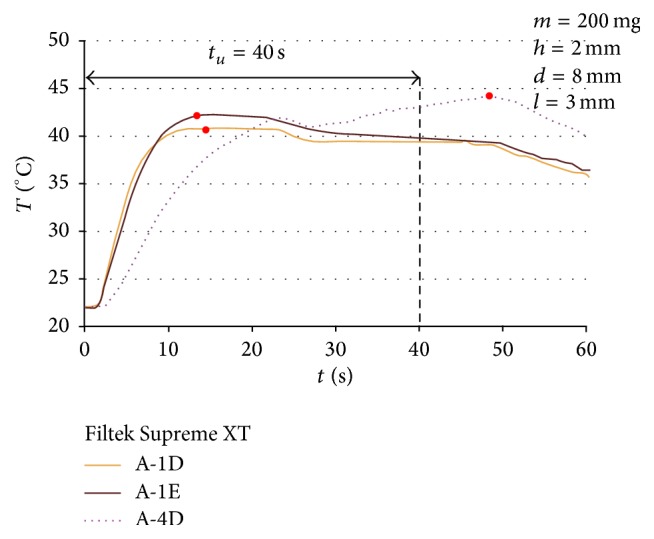
Temperature changes during the polymerization of samples of Filtek Supreme XT of various shades.

Examples of thermograms prepared at selected moments of the polymerization process of the Gradia Direct material samples (shade A-3) are shown in Figure 2. The maximum temperature values and the time of reaching them varied depending on the weight (Figure 3) and shade (Figure 4) of a given material.

**Figure 5:**
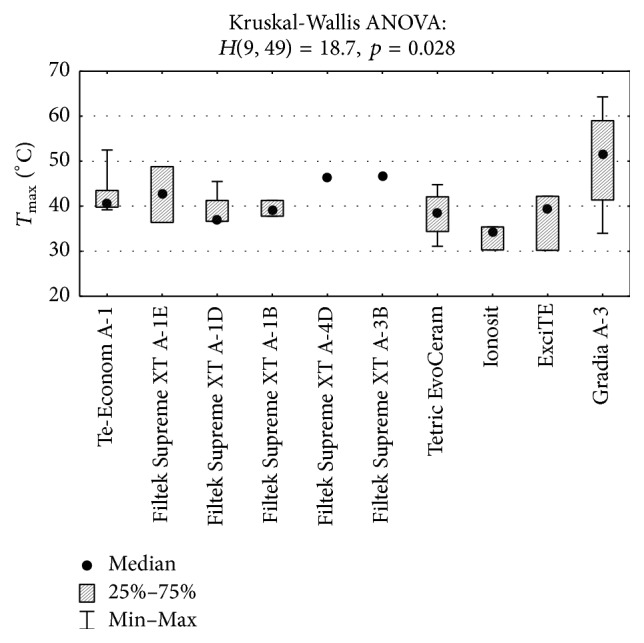
Comparison of the maximum temperature (*T*_max_) of the examined materials and the Kruskal-Wallis test result.

**Figure 6:**
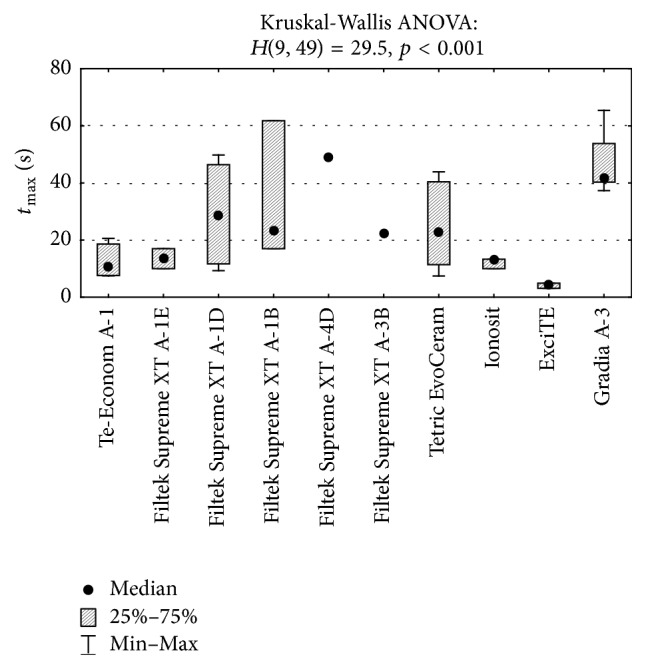
Comparison of time of reaching the maximum temperature (*t*_max_) during polymerization of the examined materials and the Kruskal-Wallis test result.

The differences between the maximum temperatures reached during polymerization by some materials were statistically significant (*p* < 0.05). The* post hoc* test (multiple comparisons) confirmed that an average temperature *T*_max_ for Gradia Direct A-3 samples was significantly higher than that for Ionosit samples (51.5 versus 34.3; *p* = 0.049) (Figure 5). The differences between the time of reaching the maximum temperatures by some materials were also statistically significant (*p* < 0.01). The* post hoc* test confirmed that an average time *t*_max_ for samples made of Gradia Direct A-3 was considerably longer than that for samples made of Te-Econom A-1 (42.0 versus 10.6; *p* = 0.009) (Figure 6) and ExciTE (42.0 versus 4.3; *p* = 0.005).

**Figure 7:**
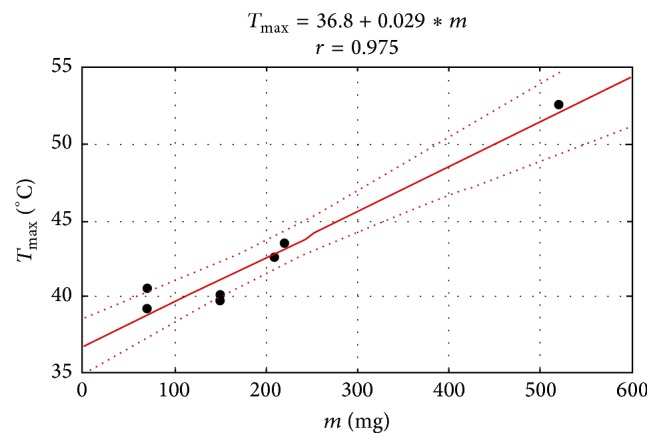
Example of a correlation diagram between the maximum temperature and the weight of a sample made of Te-Econom A-1.

A statistically significant positive correlation between the maximum temperature and the sample weight was observed (Figure 7). In some cases, placing the tip of the optic fiber at the shortest possible distance to the sample (*l* = 0) led to a dangerous increase in temperature. Placing the tip of the optic fiber in a 3 mm distance and reducing the sample weight resulted in the maximum temperature increments being reduced to safe values (Table 1).

## 4. Discussion

In all examined materials, the maximum temperature was positively correlated with the sample weight. Temperature increase and the time of reaching the maximum temperature also differed depending on the type of the material, its shade, and, to some extent, the distance from the curing unit. According to own research, samples with the Jonosit lining compomer and the ExciTE bonding agent did not cross the threshold of 42.5°C. When examining bonding agents of various generations applied in deep cavities, Khaksaran et al. [22] came to similar conclusions and they determined that the light-curing time for those materials followed in clinical practice, that is, 20 s, did not lead to any dangerous increase in temperature. It is an extremely essential conclusion, as the technique of working with a composite material requires placing the bonding agent directly on the dentine, in many cases in close proximity of the pulp.

The most considerable increase in temperature, that is, above 42.5°C, for samples weighing above 60 mg, was observed for a microhybrid material Gradia Direct. Crossing the 42.5°C threshold was also observed in the case of other examined composite materials; however, it only occurred in heavier samples of more than 200 mg. Small samples measuring around 2 × 4 mm did not reach the temperature of 42.5°C for any of the examined materials. Single-time light curing of large amount of composite in a dental cavity may result in generating heat in an amount which may be harmful for the dental pulp. It is particularly risky in the case of the first layer of the material placed in a deep cavity. Similar conclusions were drawn by Kim et al. [23]. Interesting data were gathered by Chang et al. [19] who observed a significantly higher temperature inside the sample at the depth of 1–3 mm in comparison with the deeper layers and the external surface of the material. The authors suggest that the thickness of a light-cured layer cannot exceed 3 mm, as polymerization in the deeper layers may not be sufficient due to a deficient amount of light. Therefore, in order to protect the dental pulp from overheating and, at the same time, to ensure optimum polymerization, it is crucial to apply proper volumes of light-cured materials.

The results obtained by other authors confirm the differences in the changes of temperature depending on the type of material [4, 6, 16, 17]. Al-Qudah et al. [6] demonstrated the highest increase in temperature for flowable composites, hybrid composites, compomers, and composites with increased density (condensable), respectively. In the research conducted by Hubbezoglu et al. [4], the highest temperature was observed for ormocers and flowable composites and the lowest for nanofil ones. However, in no case was the critical temperature value for pulp exceeded. According to the authors, the unique structure of every type of material, as well as the content and type of filler and resin, has a significant influence on the amount of heat generated in the course of polymerization. This thesis is supported by Dąbrowski et al. [11] who observed an increase in temperature that might be dangerous for the dental pulp during polymerization of a material which contains epoxy resins. A considerable increase in temperature may also occur in the case of recently introduced composite materials, such as bulk-fill. Their advantage is reduction of clinical application time due to a possibility of placing thicker increments. However, it involves a risk of overheating the pulp, particularly in deep, extensive cavities [17]. According to Dąbrowski et al. [10], not only the maximum increase in temperature after the light-curing of a given material but also the curing time needed for it is significant. A significant amount of heat cumulated over a short period of time may be particularly dangerous for the pulp tissue. Based on own research, considerable high values of maximum temperatures were observed in multiple heavier samples over short periods of time.

The differences between the parameters in question were also observed among various shades of the same dental material (Filtek Supreme XT). The lightest shades, A-1E (used for enamel restoration) and A-1D (for dentine restoration), reached the maximum temperature within around 14 seconds; higher temperature of 42.2°C was observed in the case of A-1E. The darker shade (A-4D-dentine) reached the maximum temperature of 49°C; however, it lasted longer, that is, 44.2 s. All the compared samples were large and they weighed 200 mg each.

Zaborowski et al. [24] obtained ambiguous results: in the case of one composite material, the most significant increase in temperature was observed for the lightest shade and for the other two materials, for the darkest one. The differences of the temperature increments that depended on the shade of the material used were also observed by other authors [20].

The increase in the temperature of a material may also depend on the light-curing unit operation mode. The authors of this study used the standard operation mode which guarantees light emission with constant intensity. According to Hubbezoglu et al. [4], the most statistically significant highest temperature was generated when using the soft-start program, and the lowest was when using the pulsation operation mode. Both light-curing variants are used in order to reduce the polymerization shrinkage of materials. However, in no case did the increase in the temperature of the dentine exceed the value which had been determined as critical for the pulp (5.5°C). Chang et al. [19], in turn, did not demonstrate any significant differences in the increase in temperatures after applying various light-curing operation modes. Changes in temperature during polymerization are also influenced by the type of equipment used. Monowave light-emitting diode generates less heat in comparison to the halogen one [2, 11, 25]. Due to the high light intensity, the new generation of LED curing units produce the same heat as QTH, and similarly to QTH, they have incorporated filters. The argon laser has been reported to be an even safer solution in this regard [26].

Therefore, it can be stated that an increase in the temperature of light-sensitive materials in the polymerization process is influenced by many factors which depend on both the material itself and the type of light-curing unit used. The amount of energy applied together with the beam of light depends on the time of exposure, the distance between the tip of the optical fiber and the material, the type of device used, the light-curing operation mode, the light intensity, and the light wavelength. The amount of heat generated in a given material depends on its composition, the thickness of the polymerized layer, and its shade [4, 6, 8, 11, 14, 18, 20, 24, 27]. Consequently, the final increase in temperature during polymerization is a derivative of many factors. In clinical practice, the thickness of the dentine layer that separates the material from the pulp is also crucial, as the dentine layer is an important protective barrier against transmitting too much heat into the pulp chamber.

## 5. Conclusions

Dentists should be aware of the potential risk involved in the use of light-sensitive dental materials. In order to minimize it, it is necessary to follow the recommendations and instructions of the manufacturers of particular dental materials and photopolymerization units, as well as to apply appropriate volumes of these materials. One needs to be particularly cautious when restoring deep cavities, as it involves the most significant risk of overheat. This process may result in posttreatment hypersensitivity or even irreversible inflammation or dental pulp necrosis.

## Figures and Tables

**Table 1:** Types of materials, sample sizes, sample maximum temperature *T*_max_, and time of reaching maximum temperature *t*_max_ (*m*: mass, *d*: diameter, *h*: height, and *l*: the distance between the polymerization unit and the sample).

Material	*m* (mg)	*h* (mm)	*d* (mm)	*l* (mm)	*T*_max_ (°C)	*t*_max_ (s)
Te-Econom A-1	70	2	4	0	40.5	7.6
	70	2	4	3	39.2	8.2
	150	5	4	0	39.8	17.5
	220	2	8	0	43.5	10.6
	520	5	8	0	52.5	20.6

Filtek Supreme XT A-1B	120	5	4	0	39.2	17.0
	490	5	8	0	41.3	23.4

Filtek Supreme XT A-3B	219	2	8	0	46.8	22.5

Filtek Supreme XT A-1D	69	2	4	0	31.4	9.3
	71	2	4	0	36.7	43.0
	123	5	4	0	37.1	49.8
	200	2	8	0	41.0	14.0
	200	2	8	3	40.8	14.4

Filtek Supreme XT A-4D	200	2	8	3	44.2	49.0

Filtek Supreme XT A-1E	68	2	4	3	36.4	10.0
	200	2	8	3	42.2	14.2

Tetric EvoCeram	69	2	4	0	31.1	22.9
	70	2	4	3	34.2	11.4
	140	5	4	0	38.5	21.8
	220	2	8	0	42.1	10.0
	210	2	8	3	39.3	43.1
	500	5	8	0	44.8	40.4

Gradia Direct A-3	60	2	4	0	36.8	44.6
	60	2	4	3	36.1	49.6
	92	5	4	0	51.6	41.4
	170	2	8	0	55.5	41.9
	172	2	8	3	62.5	42.0
	380	5	8	0	64.3	39.7

Ionosit	18	0.1	2.7	0	34.3	13.3
	19	0.1	3.0	2	35.4	12.9

ExciTE	22	0.1	6.0	0	30.2	3.1
	21	0.1	5.0	2	42.2	4.3
	22	0.1	5.5	5	39.4	4.9

E: enamel, D: dentine, and B: body.
